# Microbial exposure alters HIV-1-induced mucosal CD4^+^ T cell death pathways *Ex vivo*

**DOI:** 10.1186/1742-4690-11-14

**Published:** 2014-02-04

**Authors:** Amanda K Steele, Eric J Lee, Jennifer A Manuzak, Stephanie M Dillon, John David Beckham, Martin D McCarter, Mario L Santiago, Cara C Wilson

**Affiliations:** 1 Department of Medicine, University of Colorado Denver, Mail Stop B-168, 12700 E. 19th Avenue, Aurora, CO 80045, USA; 2 Department of Immunology, University of Colorado Denver, Aurora, CO 80045, USA; 3 Department of Surgery, University of Colorado Denver, Aurora, CO 80045, USA; 4 Department of Microbiology, University of Colorado Denver, Aurora, CO 80045, USA; 5 Department of Neurology, University of Colorado Denver, Aurora, CO 80045, USA

**Keywords:** Human Immunodeficiency Virus, Programmed cell death, Microbial translocation

## Abstract

**Background:**

Early HIV-1 infection causes massive CD4+ T cell death in the gut and translocation of bacteria into the circulation. However, the programmed cell death (PCD) pathways used by HIV-1 to kill CD4+ T cells in the gut, and the impact of microbial exposure on T cell loss, remain unclear. Understanding mucosal HIV-1 triggered PCD could be advanced by an *ex vivo* system involving lamina propria mononuclear cells (LPMCs). We therefore modeled the interactions of gut LPMCs, CCR5-tropic HIV-1 and a commensal gut bacterial species, *Escherichia coli*. In this Lamina Propria Aggregate Culture (LPAC) model, LPMCs were infected with HIV-1_BaL_ by spinoculation and cultured in the presence or absence of heat killed *E.coli*. CD4+ T cell numbers derived from flow cytometry and viable cell counts were reported relative to mock infection. Viable cells were identified by viability dye exclusion (AqVi), and intracellular HIV-1 Gag p24 protein was used to identify infected cells. Annexin V and AqVi were used to identify apoptotic versus necrotic cells. Caspase-1 and Caspase-3 activities were blocked using specific inhibitors YVAD and DEVD, respectively.

**Results:**

CD4+ T cell depletion following HIV-1 infection was reproducibly observed by 6 days post infection (dpi). Depletion at 6 dpi strongly correlated with infection frequency at 4 dpi, was significantly blocked by Efavirenz treatment, and was primarily driven by p24-negative cells that were predominantly necrotic. HIV-1 infection significantly induced CD4+ T-cell intrinsic Caspase-1 activity, whereas Caspase-1 inhibition, but not Caspase-3 inhibition, significantly blocked CD4+ T cell depletion. Exposure to *E.coli* enhanced HIV-1 infection and CD4+ T depletion, and significantly increased the number of apoptotic p24+ cells. Notably, CD4+ T cell depletion in the presence of *E.coli* was partially blocked by Caspase-3, but not by Caspase-1 inhibition.

**Conclusions:**

In the LPAC model, HIV-1 induced Caspase-1 mediated pyroptosis in bystander CD4+ T cells, but microbial exposure shifted the PCD mechanism toward apoptosis of productively infected T cells. These results suggest that mucosal CD4+ T cell death pathways may be altered in HIV-infected individuals after gut barrier function is compromised, with potential consequences for mucosal inflammation, viral dissemination and systemic immune activation.

## Background

HIV-1 infection is characterized by high levels of virus replication, gradual peripheral CD4+ T cell depletion, and aberrantly high immune activation. Chronic immune activation may result from pathogenic events in the gut mucosa established during early infection, when viral replication in the intestinal mucosa results in massive killing of lamina propria (LP) CD4+ T cells, enteropathy, inflammation, and microbial translocation [1,2]. However, the mechanisms and pathways involved in HIV-associated LP CD4+ T cell depletion remain key unanswered questions in basic HIV research [3,4].

Major efforts to investigate the mechanisms of HIV-1 mediated CD4+ T cell depletion have been made using *ex vivo* models of HIV-1 infection in primary human CD4+ T cells or cell lines. *Ex vivo* modeling studies of HIV-1 infection of primary human CD4+ T cells indicated that HIV-1-mediated killing could occur in both productively-infected and bystander, or nonproductively-infected, cells. CXCR4-tropic (X4) HIV-1 was found to kill resting spleen and tonsil CD4+ T cells *ex vivo* through abortive infection [5], whereas double-stranded DNA breaks occurring during HIV-1 integration were responsible for the death of productively-infected CD4+ T cells from peripheral blood [6]. However, it remains unclear whether the death of productively-infected or bystander cells is primarily responsible for driving human LP CD4+ T cell depletion. Interestingly, earlier studies in the SIV/rhesus macaque model also suggested that LP CD4+ T cell death could occur in both productively infected [7] and bystander [8] cells to drive depletion. Unraveling the mechanisms underlying HIV-1 mediated LP CD4+ T cell depletion may require the use of primary human LP CD4+ T cell lymphocytes.

Unlike peripheral blood or lymphoid CD4+ T cells, LP CD4+ T cells are predominantly of a recently activated, CCR5^hi^ effector memory phenotype [9]. These cells are highly susceptible to infection by CCR5-tropic HIV-1 strains, which are found in over 90% of chronically HIV-infected patients, and account for most transmitted viruses [10,11]. The LP CD4+ T cell pool in the gut-associated lymphoid tissue (GALT) is a heterogeneous population comprised of multiple T helper (Th) subsets that have diverse functions in host defense [12]. In particular, the loss of mucosal IL-17 producing T cells (Th17), which play a role in defense against extracellular pathogens, has been closely linked to pathogenic SIV and HIV infection [13-15]. The gut microbiome also plays an important role in establishing the LP microenvironment. In HIV-1 infection, translocation of microbial products strongly correlates with increased immune activation [16-18]. In fact, commensal Gram-negative *Escherichia coli* (*E.coli*) was detected in the LP of rhesus macaques during early stages of pathogenic SIV infection [19]. Thus, adequately modeling HIV interactions in the LP must account for any effects of commensal bacteria. We previously showed that commensal *E.coli* activates resident LP CD4+ T cells in an MHC Class II-dependent fashion [20], and increases HIV-1 replication in human LP CD4+ T cells *ex vivo*[21]. However, the impact of microbial exposure on the magnitude and mechanisms of LP CD4+ T cell death remains unknown.

To date, very limited information exists as to which cell death pathway(s) are triggered in human LP CD4+ T cells by R5-tropic HIV-1 infection. Most programmed cell death (PCD) pathways are dependent on proteolytic enzymes known as caspases. In the canonical PCD pathway, apoptosis, cells undergo cell shrinkage, blebbing and DNA fragmentation but retain plasma membrane integrity [22]. Apoptosis can be triggered by either extrinsic (e.g. Fas/FasL) or intrinsic stimuli, but both pathways converge on the effector molecule Caspase-3 [23]. Apoptotic cells are generally disposed of *in vivo* in a non-inflammatory manner through the exposure of phosphotidylserine (PS) from the inner leaflet of the plasma membrane to the cell surface [24-26]. There is a substantial body of literature suggesting that apoptosis is aberrantly triggered or has become dysregulated during HIV-1 infection [27-29]. In contrast to apoptosis, pyroptosis is a highly inflammatory form of PCD that involves oncosis, plasma membrane rupture, and the rapid release of cytoplasmic contents into the surrounding environment [22,30]. Pyroptosis has been linked to the ‘inflammasome’, a multimeric complex containing active Caspase-1 and pattern recognition receptors such as NLRP3 [30,31]. In addition to mediating pyroptosis, Caspase-1 processes pro-IL-1β to the mature secreted form that could contribute to inflammation and epithelial barrier dysfunction [32,33]. Interestingly, increased Caspase-1 activity has been documented in HIV infection *ex vivo* in the Human Lymphoid Aggregate Culture (HLAC) model and in primary peripheral blood T cells from HIV infected patients [5,34,35]. It remains unknown whether Caspase-1 plays a role in HIV-1 mediated LP CD4+ T cell death.

In this report, we used the Lamina Propria Aggregate Culture (LPAC) model to identify the PCD pathway(s) triggered in primary LP CD4+ T cells *ex vivo* by infection with an R5 tropic HIV-1 strain. We further assessed the impact of commensal *E. coli* exposure on the magnitude and mechanisms of HIV-mediated LP CD4+ T cell depletion. We provide evidence for augmented HIV-1 mediated LP CD4+ T cell death and a shift in the PCD pathway following microbial exposure.

## Results

### CD4+ T cell depletion strongly correlates with productive infection in the LPAC model

**Figure 1:**
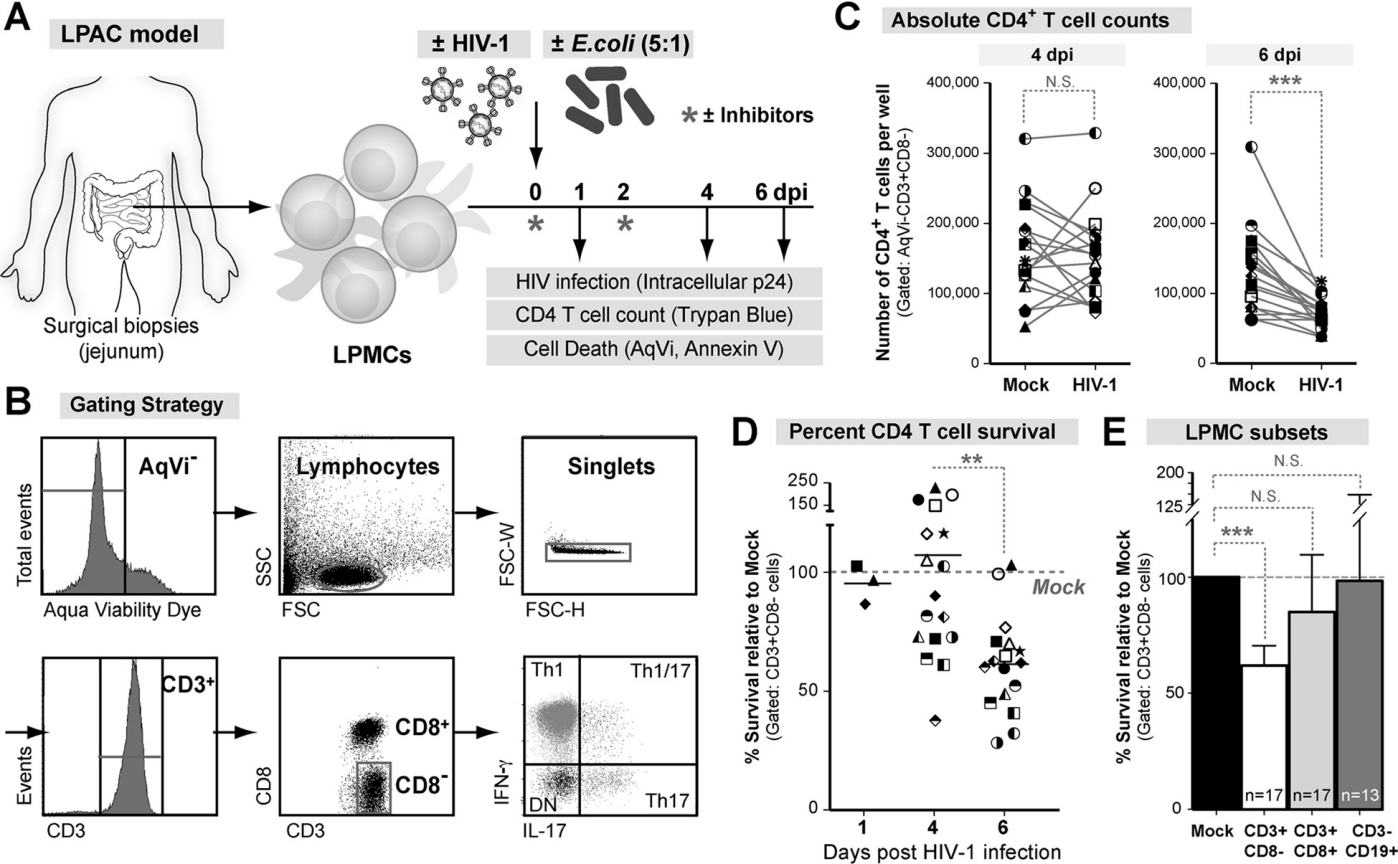
**HIV-1 mediated cell death can be quantified in the LPAC model. (A)** Lamina Propria Aggregate Culture (LPAC) model. Primary LPMCs from discarded surgical samples were infected with CCR5-tropic HIV-1_Ba-L_ (80 ng p24 per 1 M LPMC), cultured for 1–6 days and harvested for flow cytometry. **(B)** Representative gating strategy. **(C)** The absolute number of CD4+ T cells per well in mock or HIV-1 infected conditions. N = 17 donors. Each symbol is a unique donor. Significant differences at each time point was determined by Wilcoxon matched pairs signed rank test. **(D)** To compare relative depletion between donors, CD4+ T cell survival was normalized to mock infection (100% survival). Each symbol is an individual donor. Horizontal solid lines reflect the median. Significance was determined using the Kruskal-Wallis test across timepoints. **(E)** Cell-specificity of HIV-1 mediated depletion in the LPAC model. The percent survival relative to mock infection at 6 dpi was determined for CD3+CD8- T cells (white), CD3+CD8+ T cells (light grey), and CD3-CD19+ B cells (dark grey). The black bar depicts 100% survival for each cell type in the mock infected condition, also indicated by the dashed horizontal line. Significance was determined using the Kruskal-Wallis test and Dunn’s multiple comparison test to compare selected pairs of columns. **p = 0.0015; ***p = 0.0003; ****p < 0.0001.

To monitor HIV-1 mediated LP CD4+ T cell death, we utilized primary LP mononuclear cells (LPMC) in an experimental strategy which involved quantifying absolute LP CD4+ T cell numbers in HIV-1-infected relative to mock infected cultures [5]. Given the similarities of our experimental approach to that of the HLAC model, we refer to this infection platform as the ‘Lamina Propria Aggregate Culture’ or LPAC model. LPMCs were infected with R5-tropic HIV-1_Ba-L_ by spinoculation and analyzed at the indicated days post-infection (dpi) (Figure 1A). Since HIV-1 downregulates surface CD4 expression *in vitro*[36], CD4+ T cells were identified as CD3+CD8-. Viable CD3+CD8- T cells were identified using the gating strategy shown (Figure 1B). At 4 dpi, the absolute numbers of CD4+ T cells in mock infected and HIV-infected wells were not significantly different (Figure 1C *left panel*). However, by 6 dpi, HIV-1-infected wells had significantly fewer CD4+ T cells than mock (Figure 1C *right panel)*. To compare the extent of HIV-1-mediated depletion between donors, the survival of CD3+CD8- T cells was normalized to mock (100% survival). Depletion was not statistically significant overall at 4 dpi, but about half (9 of 17) of the samples exhibited some level of depletion (‘depleters’; Figure 1D). Depletion was significantly greater at 6 dpi than at 4 dpi (Figure 1D). CD3+CD8+ T cells and CD3-CD19+ B cells were not depleted relative to mock infection (Figure 1E). Thus, LP CD4+ T cell depletion can be specifically and reliably quantified in the LPAC model by 6 dpi.

**Figure 2:**
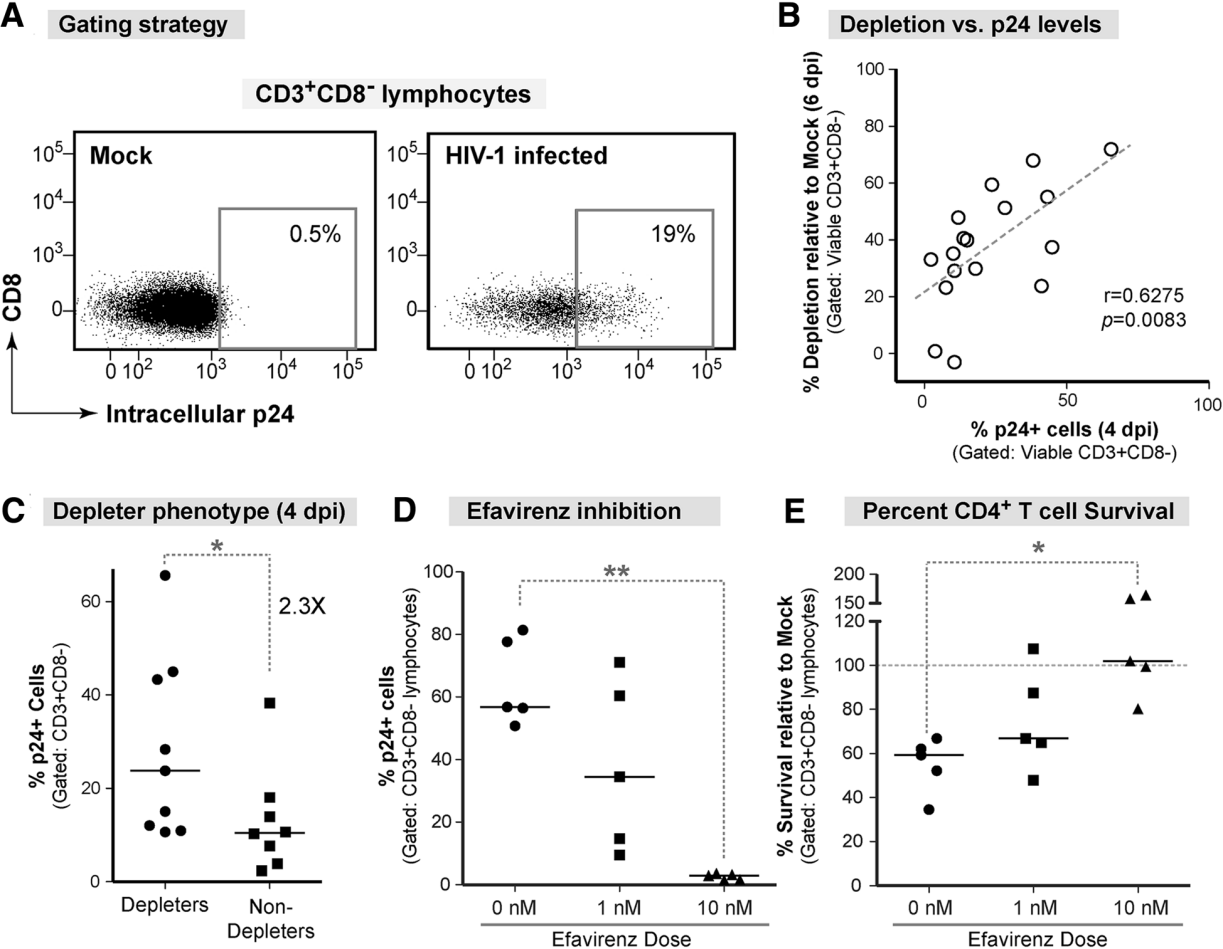
**Relationship between HIV-1 infection and LP CD4+ T cell depletion. (A)** Representative flow plots identifying p24+ cells using the gate established in mock infection (<1%). The net frequency of p24+CD4+ T cells (e.g.,%p24+ =%p24+ in HIV-1 minus %p24+ in Mock) is reported. **(B)** Spearman rank correlation analysis between the percentage of p24+ cells at 4 dpi and the level of LP CD4+ T cell depletion at 6 dpi. The best-fit line, Spearman *r* and the *p* value are indicated. **(C)** The percentage of p24+ cells was compared between ‘depleters’ and ‘non-depleters.’ Each point is a unique sample. The horizontal solid line indicates the median infection level. Statistical significance was determined by Mann–Whitney U test, *p = 0.03. **(D-E)** LPMCs were pre-treated with Efavirenz for 2 h prior to infection with HIV-1_Ba-L_ or mock and re-dosed at 3 dpi. The net frequencies of p24+ cells (overall ANOVA *p =* 0.0008) were calculated for each donor at 6 dpi (Figure 2D). The percentage of CD4+ T cells surviving relative to mock infection in the presence of Efavirenz is shown (overall ANOVA *p =* 0.0394) (Figure 2E). The medians are shown and the dashed horizontal line indicates 100% survival relative to mock infection. Statistically significant differences were determined using the Friedman Test and Dunn’s Multiple Comparison test to compare between groups.

We then assessed the relationship between productive HIV-1 infection and LP CD4+ T cell depletion. Productive HIV-1 infection was measured using intracellular HIV-1 Gag-p24 (p24) expression. Positive p24 expression was established from the mock infected control (<1% p24+) (Figure 2A). The frequency of CD3+CD8-p24+ cells at 4 dpi strongly correlated with LP CD4+ T cell depletion at 6 dpi in HIV-infected cultures (Figure 2B). In addition, the percentage of p24+ cells was 2.3-fold greater in the 9 ‘depleter’ samples (Figure 1D) than the 8 ‘non-depleter’ samples (Figure 2C) despite similar cellular LPMC composition prior to infection (Additional file 1: Figure S1). To confirm that depletion was dependent on productive HIV-1 infection, early reverse transcription was inhibited using the antiretroviral drug, Efavirenz. Efavirenz at 1 nM or 10 nM blocked p24 expression and T cell depletion in a dose dependent fashion (Figure 2D). The 10 nM Efavirenz dose was sufficient to significantly decrease p24 expression and increase the survival of CD3+CD8- T cells relative to untreated HIV infection (Figure 2E). These results indicate that in the LPAC model, active HIV-1 replication is required for LP CD4+ T cell depletion.

### HIV-1 infection increases the frequency of CD4 T cells with apoptotic and necrotic phenotypes

**Figure 3:**
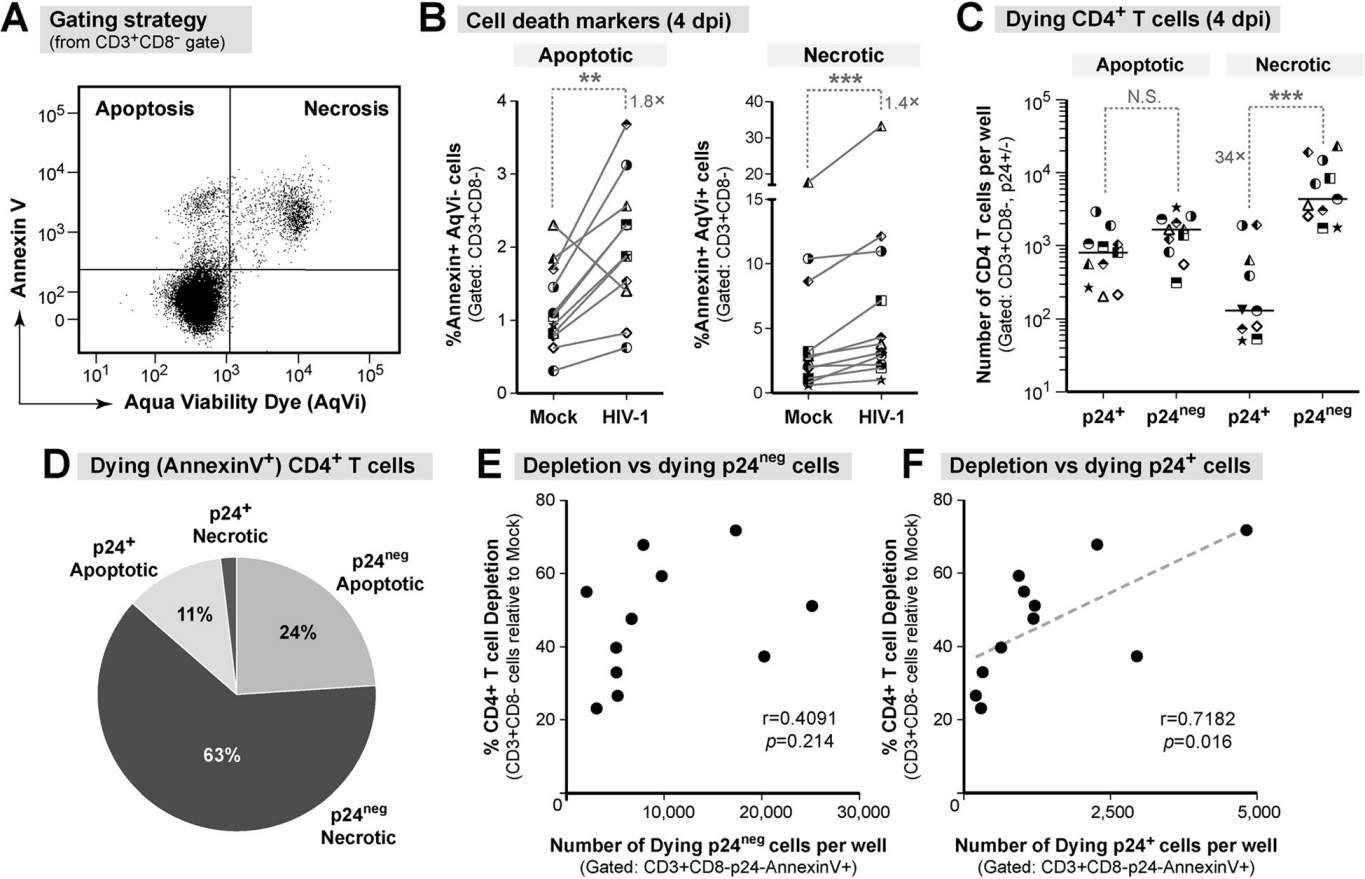
**HIV-1 infection increases cell death markers in LP CD4+ T cells. (A)** Representative flow plot of AnnexinV/AqVi staining. Note that in contrast to Figure 1B, there was no AqVi exclusion gate prior to identifying lymphocytes from the FSC/SSC profile. Cells expressing AnnexinV were considered committed to dying. **(B)** The percentage of CD3+CD8- T cells expressing either an apoptotic (*left*) or necrotic (*right*) profiles were compared between mock and HIV-1 infected conditions. Each symbol is a unique donor. The fold differences in median expression are shown. Significance was determined using the Wilcoxon matched-pairs signed rank test. **(C)** The numbers of p24+ and p24^neg^ cells per well with an apoptotic or necrotic phenotype were calculated. Each symbol represents a unique donor. The median values are shown and the fold-change is indicated for significant differences. Significance was determined using the Wilcoxon matched-pairs signed rank test. **(D)** Apoptotic versus necrotic p24+ and p24^neg^ cells as a percentage of the total number of LP CD3+CD8- T cells committed to death (n = 11). **(E-F)** Spearman rank correlation analysis between the number of AnnexinV+ **(E)** p24^neg^ and **(F)** p24+ cells 4 dpi versus the 6 dpi depletion levels. The best-fit lines, Spearman *r* and *p* values are indicated. **, *p* = 0.009; ***, *p* = 0.001.

To address potential mechanisms of HIV-1-mediated LP CD4+ T cell death, we profiled the CD4+ T cells either as early apoptotic (AnnexinV+AqVi-) or necrotic (AnnexinV+AqVi+) by flow cytometry (Figure 3A). Multiple PCD pathways, including apoptosis that has proceeded to secondary necrosis, pyroptosis, and necroptosis, converge on the necrotic phenotype given sufficient time [24,25,37]. Cell death profiles were measured at 4 dpi when early apoptotic cells were detectable. HIV-1 infection increased the percentage of CD4+ T cells expressing both apoptotic and necrotic phenotypes relative to mock (Figure 3B).

We next compared the p24+ and p24^neg^ populations to gain insight into their respective contributions to total CD4+ T cell loss. The percentage of cells with an apoptotic phenotype was greater in the p24+ population, whereas the percentage of cells with a necrotic phenotype was greater in p24^neg^ population (Additional file 2: Figure S2A). However, since there were more p24^neg^ cells than p24+ cells in the culture, the absolute numbers of apoptotic p24+ and p24^neg^ cells were similar at 4 dpi (Figure 3C), suggesting that apoptosis contributed equally to depletion in both subsets. In sharp contrast, the absolute number of p24^neg^ cells with a necrotic phenotype was 34-fold greater than the number of necrotic p24+ cells (Figure 3C). Presented another way, 63% of the CD4+ T cells committed to dying (AnnexinV+) were p24^neg^ and had a necrotic phenotype (Figure 3D), indicating that necrotic cell death in the p24^neg^ population accounted for the majority of depletion during HIV-1 infection. The p24+ cells accounted for only 13% of the total dying cells (Figure 3D), but it was the magnitude of p24+ cell death that correlated with HIV-1 mediated LP CD4+ T cell depletion measured at 6 dpi (compare Figure 3E and F).

### HIV-1 promotes LP CD4+ T cell death predominantly through Caspase-1-mediated pyroptosis

**Figure 4:**
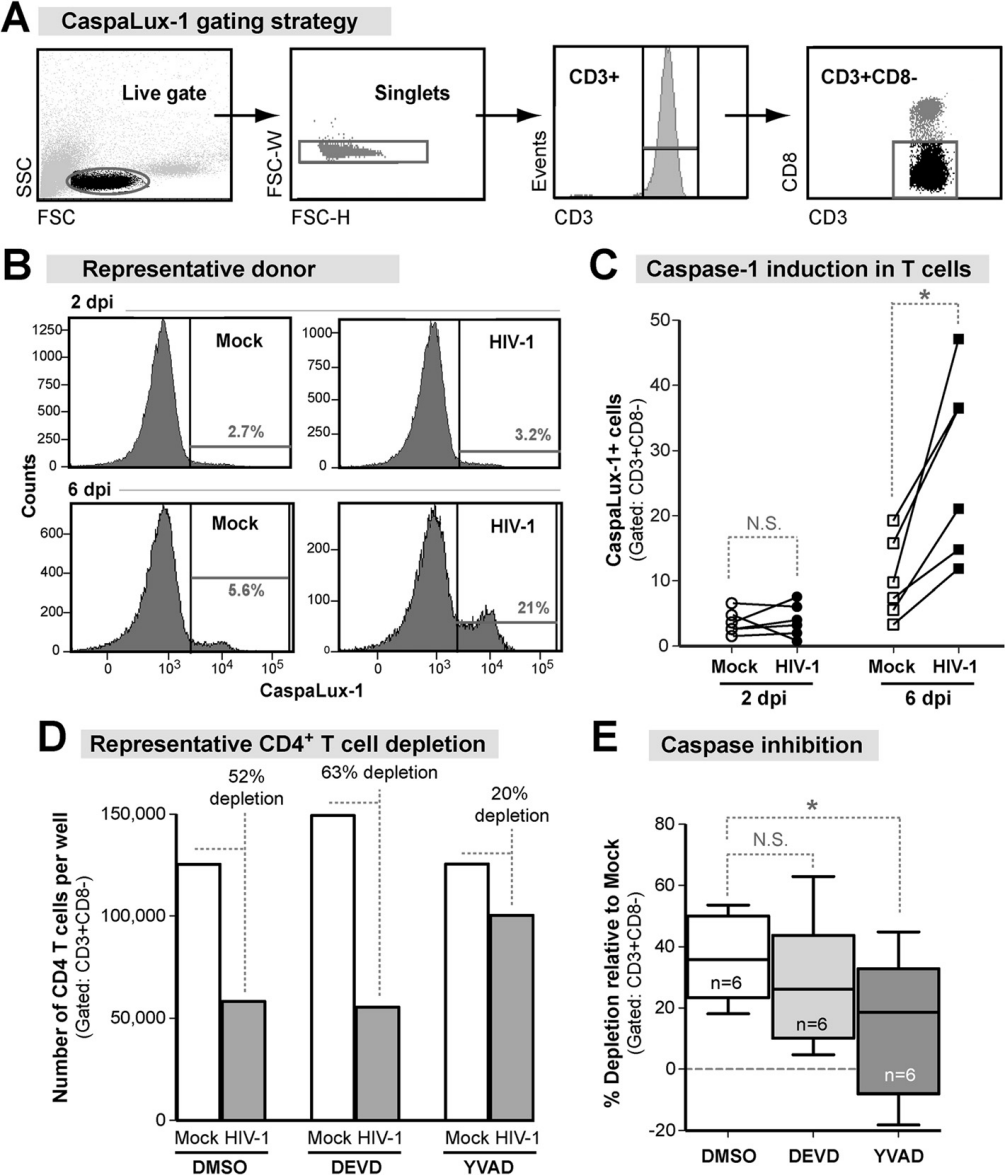
**HIV-1 induced T cell Caspase-1 activity and death in non-productively infected LP CD4+ T cells. (A)** Detection of active Caspase-1 in T cells. At 2 and 6 dpi, LPMCs were harvested and incubated for 30 min with the CaspaLux-1 Substrate (Oncoimmunin) then surface stained to allow for identification of CD3+CD8- T cells by flow cytometry. Cells were gated from a tight lymphocyte gate that excluded cellular debris. Cleaved CaspaLux-1-E_1_D_2_ was detected in CD3+CD8- T cells using the FITC channel. **(B)** Representative histograms of Caspalux-1-E_1_D_2_ staining. **(C)** HIV-1 induced Caspase-1 activity in LP CD4+ T cells. The fold change in median Caspase-1 activity is shown at 6 dpi. Significance was determined using the Wilcoxon matched-pairs signed rank test. **(D)**. YVAD (Caspase-1; 25 μM), DEVD (Caspase-3; 25 μM) or a DMSO vehicle control were added at 0 and 2 dpi and CD4+ T cell survival was evaluated at 4 dpi. LP CD4+ T cell survival was normalized to parallel mock infections. The number of cells per well in a representative donor with mock (white bar) or HIV-1 (grey bar) infection with DMSO (*left*), DEVD (*center*) and YVAD (*right*). The percent depletion from mock are indicated for each condition. **(E)** The percent depletion from mock infected controls are shown in the presence of DMSO (white box), DEVD (light grey), and YVAD (dark grey) (n = 6). Box and whiskers indicate the median and range respectively. Significant differences in the percent depletion from the DMSO vehicle were determined using non-parametric repeated measures ANOVA. Dunn’s multiple comparison tests were used to compare selected pairs of columns as shown. Overall ANOVA, *p* = 0.0003. *p = 0.02–0.03.

We next considered non-apoptotic PCD pathways that could lead to a necrotic phenotype. Pyroptosis is a Caspase-1-dependent PCD pathway that was recently implicated in HIV-1-mediated death of resting lymphoid T cells [5]. Caspase-1 activity was measured by flow cytometry in mock or HIV-infected LPMCs at 2 and 6 dpi using a fluorogenic Caspase-1 substrate (CaspaLux-1). CD3+CD8- T cells with active Caspase-1 were identified from a tight lymphocyte gate (Figure 4A-B). Caspase-1 activity was significantly induced in LP CD4+ T cells in HIV-infected cultures compared to mock by 6 dpi (Figure 4C). These findings provide evidence for LP CD4+ T cell intrinsic Caspase-1 activity that occurred concurrent with depletion.

Irreversible peptide inhibitors were used to block the function of Caspase-1 (pyroptosis; YVAD) and Caspase-3 (apoptosis; DEVD) to determine their relative effects on HIV-1 induced depletion. To avoid toxicity, we used the highest possible dose (25 μM) of each inhibitor at a non-toxic DMSO concentration and added each inhibitor (25 μM) at days 0 and 2 (Figure 1A). Depletion was determined relative to mock infection and normalized to the DMSO vehicle control (100% depletion) to determine the percent protection from depletion with each inhibitor for each donor (Figure 4D; representative donor). Neither DEVD nor YVAD significantly altered the frequency of p24+ CD4+ T cells compared to the DMSO control, indicating that HIV-1 replication was not impeded by the presence of drug (Additional file 3: Figure S3A). In 6 donors, HIV-1 infection significantly depleted LP CD4+ T cells by 4 dpi relative to mock infection with DMSO (Figure 4E). DEVD did not significantly inhibit LP CD4+ T cell depletion (Figure 4E). However, YVAD significantly attenuated HIV-1 mediated depletion from 36% to 18% (Figure 4E) indicating a role for Caspase-1 activity in HIV-1 mediated LP CD4+ T cell death.

### Exposure to commensal *E. coli* alters the kinetics and increases the magnitude of HIV-1 mediated LP CD4+ T cell depletion in the LPAC model

**Figure 5:**
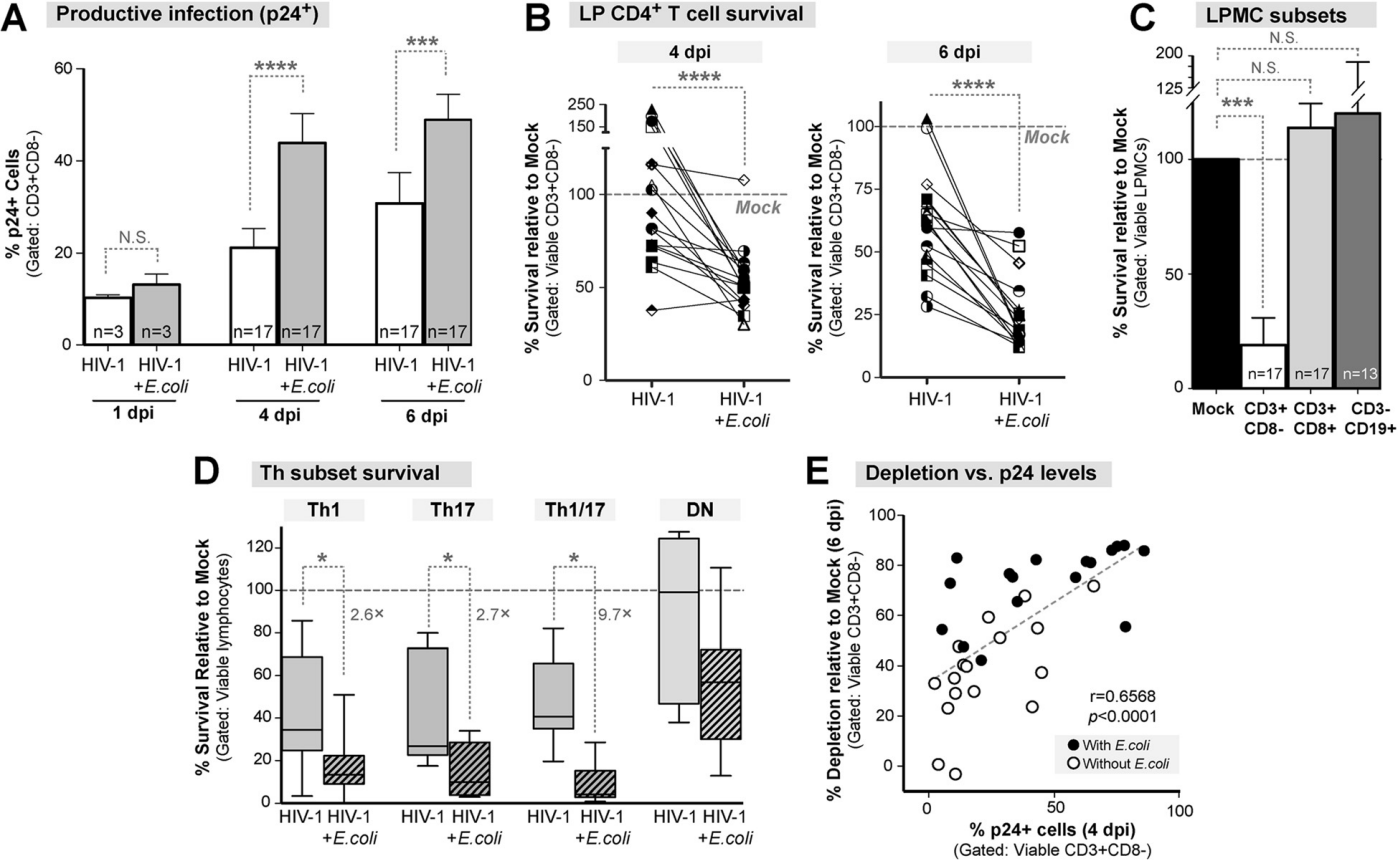
**Commensal *E. coli *enhance apoptosis in productively infected LP CD4+ T cells. (A)** Commensal *E. coli* increases the frequency of productively infected cells. The percentage of p24+ cells in the presence of a 5:1 *E. coli: LPMC* ratio was quantified using intracellular p24 expression. Significance was determined using the Wilcoxon matched-pairs signed rank test. **(B)** LP CD4+ T cell survival was compared in the presence of absence of *E. coli* normalized to mock (100% survival). Significant differences in cell survival at 4 dpi or 6 dpi were determined using the Wilcoxon matched-pairs signed rank test. **(C)** Cell-specificity of HIV-1 mediated depletion in the LPAC model. The percent survival relative to mock infection at 6 dpi was determined for CD3+CD8- T cells (white), CD3+CD8+ T cells (light grey), and CD3-CD19+ B cells (dark grey). The black bar depicts 100% survival for each cell type in the mock infected condition, also indicated by the dashed horizontal line. Significance was determined using the Kruskal-Wallis test and Dunn’s multiple comparison tests to compare selected pairs of columns. **(D)** Th Subset survival was determined following stimulation with PMA/Ionomycin. Viable cell counts were obtained prior to PMA/Ionomycin and multipled by the percentage of CD3+CD8- T cells expressing cytokine. Survival was determined normalized to mock infection for all subsets. Box and whiskers indicate the median and range respectively. Significant differences in cell survival for each subset were determined using the Wilcoxon matched-pairs signed rank test. **(E)** Spearman rank correlation analysis between the percentage of p24+ cells at 4 dpi and the level of LP CD4+ T cell depletion at 6 dpi. The best-fit line, Spearman *r* and the *p* value are indicated. *p = <0.05; ***p = 0.0005; ****p < 0.0001.

We previously reported that commensal *E. coli* exposure augmented HIV-1 infection of LPMCs [21], but the impact of *E. coli* exposure on LP CD4+ T cell death remained unknown. As expected, *E. coli* exposure in the LPAC model significantly increased the frequency (Figure 5A) and number (data not shown) of p24+ cells at 4 dpi and 6 dpi relative to HIV-1 infection only. Importantly, LP CD4+ T cell depletion was also significantly enhanced at both 4 dpi (Figure 5B *left panel*) and 6 dpi (Figure 5B *right panel*) in the presence of *E. coli*. This contrasts with the kinetics of depletion in the HIV-1 infection without *E. coli* condition, when reproducible depletion was not observed until 6 dpi (Figure 1D). Of note, no depletion was observed in CD3+CD8+ or CD3-CD19+ cells in the presence of *E. coli* (Figure 5C), indicating that the enhanced depletion was specific to CD4+ T cells and not due to potential non-specific cell death from direct microbial activation. In summary, exposure to commensal *E. coli* increased the magnitude and kinetics of LP CD4+ T cell depletion in the LPAC model.

Pathogenic lentivirus infection is associated with extensive depletion of the gut Th17 subset which plays a key role in bacterial defense [13,14,38-40], raising the possibility that the Th subsets in the LP may have different susceptibilities to HIV-1 mediated killing. We therefore investigated the susceptibility of Th1 (IFN-γ+IL-17-), Th17 (IL-17+IFN-γ-), Th1/17 (IFN-γ+ IL-17+) and non-Th1/17 (IFN-γ-IL-17-, double negative, DN) subsets to undergo HIV-1 mediated death at 6 dpi in the presence or absence of bacteria. The Th subsets were identified by intracellular cytokine staining by flow cytometry following PMA/Ionomycin stimulation. At 6 dpi, R5-tropic HIV-1 depleted Th1, Th17 and Th1/17 cells in the LPAC model to a similar extent (Figure 5D). In contrast, DN cells were relatively resistant to HIV-1 mediated killing (Figure 5D). Commensal *E. coli* exposure led to 2 to 3-fold increased LP T cell depletion in the Th1, Th17 and DN subsets compared to HIV-1 alone (Figure 5D). Remarkably, *E. coli* enhanced depletion in the Th1/17 depletion by 10-fold (Figure 5D). As with HIV-1 infection alone (Figure 2B), the level of productive infection was strongly associated with the level of depletion following *E. coli* exposure (Figure 5E; Spearman *r* for samples with *E.coli* = 0.5760; *p =* 0.017).

### Exposure to *E. coli* increases the death of productively infected cells through increased apoptosis

**Figure 6:**
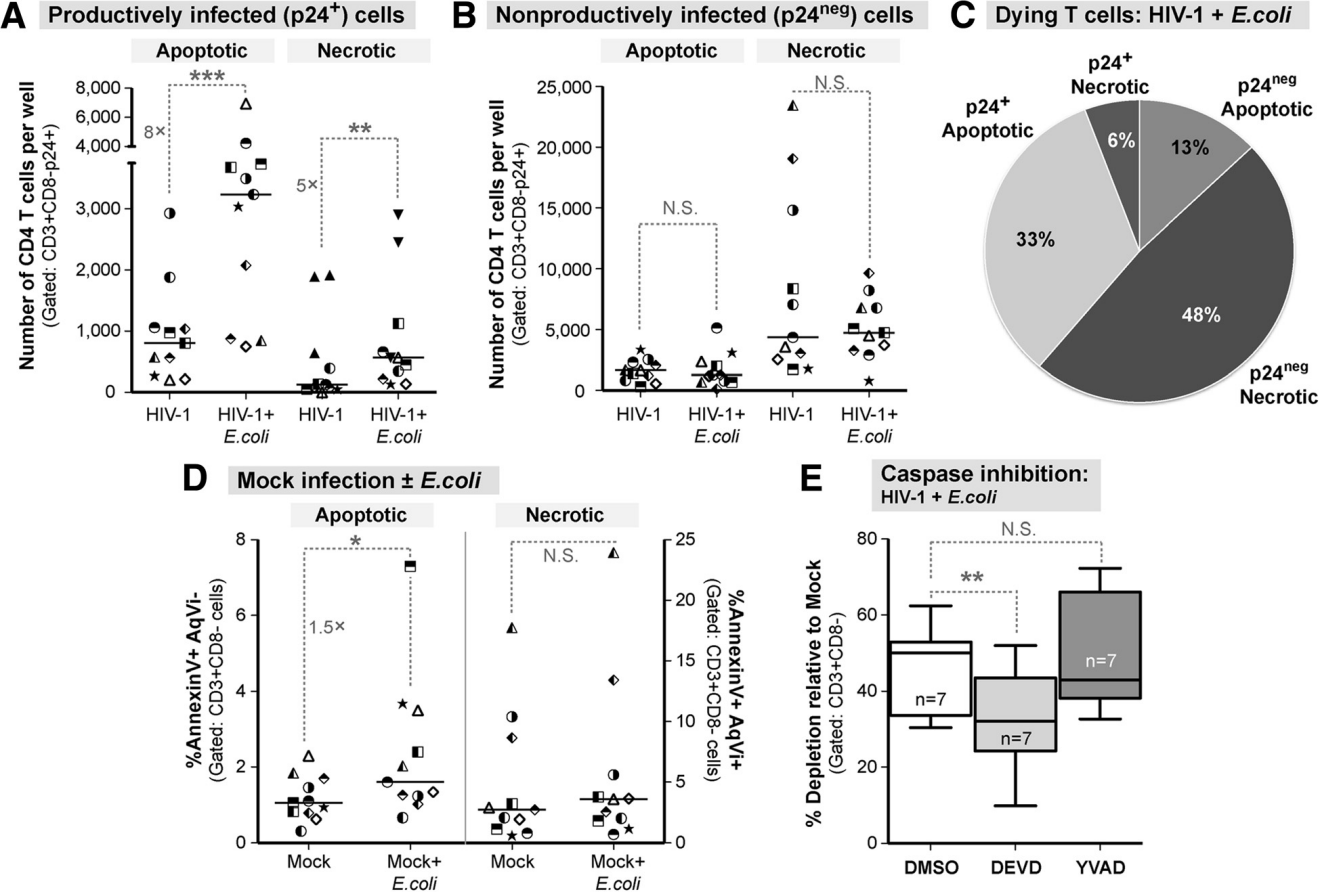
***E. coli *alters caspase-dependent LP CD4+ T cell death. (A and B)** Impact of *E.coli* exposure on apoptotic and necrotic phenotypes in the LPAC model. The absolute numbers of p24+ cells that were either apoptotic (*left*) or necrotic (*right*) were determined as in Figure 3 for **(A)** p24+ and **(B)** p24^neg^ cells. Each symbol represents a unique donor. Significance was determined using the Wilcoxon matched-pairs signed rank test within each phenotype. **(C)** Apoptotic versus necrotic p24+ or p24^neg^ cells as a percentage of the total LP CD3+CD8- T cells committed to death (n = 11). Compare to Figure 4B. **(D)** Impact of *E.coli* on cell death markers in uninfected LPMCs. The percentage of apoptotic or necrotic mock-infected cells were compared with or without *E.coli*. The horizontal lines indicate the median values and the fold-differences in cell death marker expression. Significance was determined using the Wilcoxon matched-pairs signed rank test within each phenotype. **(E)** The percent depletion from mock infection in the presence of YVAD and DEVD (n = 7) as described in Figure 5 in the presence of *E.coli*. Overall ANOVA, *p* = 0.008. *p = < 0.05 **, *p* = 0.007; ***, *p* = 0.001.

Since *E. coli* exposure increased the number of p24+ cells and overall depletion, we assessed the proportion of cells with an early apoptotic or necrotic phenotype in the p24+ and p24^neg^ populations (Figure 3A) in the presence of *E. coli.* As with HIV-1 infection alone, p24+ cells remained more likely to have an apoptotic phenotype than p24^neg^ cells, whereas p24^neg^ cells remained more likely to have a necrotic profile than p24+ cells (Additional file 2: Figure S2B). Notably, the absolute number of p24+ cells with both apoptotic and necrotic phenotypes increased significantly in the presence of *E. coli* compared to HIV-1 alone (Figure 6A). In contrast, *E. coli* had no effect on the absolute number of p24^neg^ cells with either an apoptotic or necrotic phenotype (Figure 6B). In total, p24+ cells accounted for 39% of the CD4+ T cell death in the presence of *E. coli* (Figure 6C), in sharp contrast to HIV-1 infection only (Figure 3D). Importantly, the majority of the p24+ CD4+ T cells were apoptotic in the presence of *E. coli*. The addition of *E. coli* significantly increased the percentage of cells with an apoptotic phenotype by 1.5-fold in mock infected cells (Figure 6D) but did not alter the percentage of cells with a necrotic phenotype (Figure 6D). Thus, increased HIV-1 mediated LP CD4+ T cell apoptosis in the presence of *E.coli* likely reflects both virus- and bacteria-induced cell death.

DEVD and YVAD were used to determine whether Caspase inhibition could block HIV-1 associated LP CD4+ T cell death in the presence of *E. coli*. The inhibitors did not significantly decrease the LP CD4+ T cell number in the mock plus *E. coli* condition (Additional file 3: Figure S3C-D). LP CD4+ T cell depletion in the presence of HIV-1 plus *E. coli* was significantly blocked by DEVD compared to the DMSO control (Figure 6E). Surprisingly, Caspase-1 inhibition by YVAD did not significantly prevent LP CD4+ T cell depletion with virus and *E. coli* present (Figure 6E). The results suggest that in the presence of *E.coli,* HIV-1 mediated LP CD4+ T cell death is Caspase-3, but not Caspase-1, dependent.

## Discussion

Counteracting inflammation and immune activation in HIV-1 infection may require resolving critical knowledge gaps about how HIV-1 causes extensive LP CD4+ T cell death during primary infection. Here, we demonstrate the versatility of the LPAC model in addressing these critical questions regarding the mechanisms underlying R5-tropic HIV-1 mediated mucosal CD4+ T cell death. We provide evidence that HIV-1 induces pyroptosis of non-productively infected LP CD4+ T cells based on the detection of active Caspase-1 in CD4+ T cells and the inhibition of LP CD4+ T cell depletion by blocking Caspase-1 activity. Surprisingly, our results with primarily effector memory LP CD4+ T cells mirrored published findings based on X4-tropic HIV-1 infection of HLAC cells [5], which are predominantly resting CD4+ T cells. Thus, despite the important differences in CD4+ T cell phenotypes between the two *ex vivo* models, pyroptosis appears to be a critical HIV-1-induced PCD pathway in non-productively infected CD4+ T cells.

The molecular trigger(s) for Caspase-1 mediated death in the LPAC model remain unknown, but recent high-profile studies suggest plausible mechanisms [5,6,41,42]. One possibility is that the accumulation of incomplete HIV-1 reverse transcripts in abortively infected LP CD4+ T cells triggered death, analogous to that reported in the HLAC model [5]. In this model, IFI16 has been identified as the DNA sensor that triggers pyroptosis in the bystander T cells [42], and p24+ cells were shown to be the killing unit rather than the free virus [5]. Notably, in the LPAC model, it was the number of dying p24+, but not p24^neg^ cells, which significantly correlated with depletion. If a similar PCD mechanism operates in the LPAC as in the HLAC model, each p24+ cell could induce cell death in variable number of bystander cells, thereby weakening the association between p24^neg^ cells and depletion. However, LP CD4+ T cells are more susceptible to HIV-1 infection than resting HLAC CD4+ T cells due to higher activation levels and expression of HIV-1 co-receptors [9,43]. Thus, at this time, we cannot confirm that abortive infection triggers pyroptosis in the gut and cannot exclude alternative triggers for Caspase-1 activity. Nabel and colleagues recently reported decreased HIV-1 gene expression following DNA-PK recognition of double-stranded DNA breaks during integration [6]. Thus, an alternative hypothesis is that HIV-1 gene expression is being quickly shut off in permissive LP CD4+ T cells upon integration induced cell death, and permissive cells dying post-HIV-1 integration could appear as p24^neg^. Loss of viral protein expression in permissive cells could also potentially explain the strong association we observed between the frequency of productively infected cells and depletion. However, to our knowledge, there is no established link between DNA-PK and Caspase-1 activities.

Caspase-1 activity may be a potential link between HIV-1-mediated LP CD4+ T cell death and HIV-1-mediated mucosal inflammation. In antigen presenting cells, Caspase-1 cleaves pro-IL-18 and pro-IL-1β to their active forms [30]. Increased IL-1β expression was observed in the LP during acute pathogenic SIV, but not during non-pathogenic infection, suggesting that Caspase-1 activity could be a mediator of lentivirus pathogenesis [38]. IL-1β, other pro-inflammatory cytokines, and HIV-1 gp120 have been shown to cause tight junction breakdown between intestinal epithelial cells *in vitro*[32,44]. Furthermore, pyroptosis releases cellular contents into the environment that can act as danger-associated molecular patterns (DAMPs), thereby triggering inflammation in the local microenvironment [30,31]. Thus, Caspase-1 activity in LP CD4+ T cells could precipitate epithelial barrier dysfunction by releasing either traditional inflammatory cytokines or cellular DAMPs.

Epithelial barrier breakdown results in microbial translocation during the later stages of acute SIV infection [19], suggesting that studies in the LPAC model are incomplete without taking enteric bacteria into account. We previously reported [21] that exposure to commensal bacteria increased productive infection in LP CD4+ T cells *in vitro*, likely by enhancing T cell activation. We now link these findings to increased LP CD4+ T cell depletion in the presence of commensal bacteria. In addition, the LPAC data for Th subsets recapitulated the high susceptibility of IL-17-producing cells to HIV-1-mediated depletion observed *in vivo* and highlighted a role for microbial exposure in exacerbating this process [13,14,38-40].

Exposure to *E. coli* increased overall LP CD4 T cell depletion by increasing the number of productively infected cells dying by apoptosis. The involvement of apoptosis as the dominant PCD pathway in the presence of *E. coli* was further supported by significant inhibition of HIV-1-mediated depletion by blocking Caspase-3 activity. Several viral proteins such as protease, gp120, Tat, Nef and Vpr have been reported to facilitate apoptotic CD4+ T cell death [27,28]. Enhanced HIV-1 infection following microbial exposure could lead to increased cellular levels of these viral proteins, thereby triggering apoptosis. Indirectly, microbial products may also sensitize LP CD4+ T cells to undergo apoptosis at a lower signaling threshold. For example, LPS and inflammatory cytokines have been reported to increase tryptophan catabolism by DCs [15], potentially causing local tryptophan depletion, which then sensitizes activated T cells to apoptosis [45,46].

**Figure 7:**
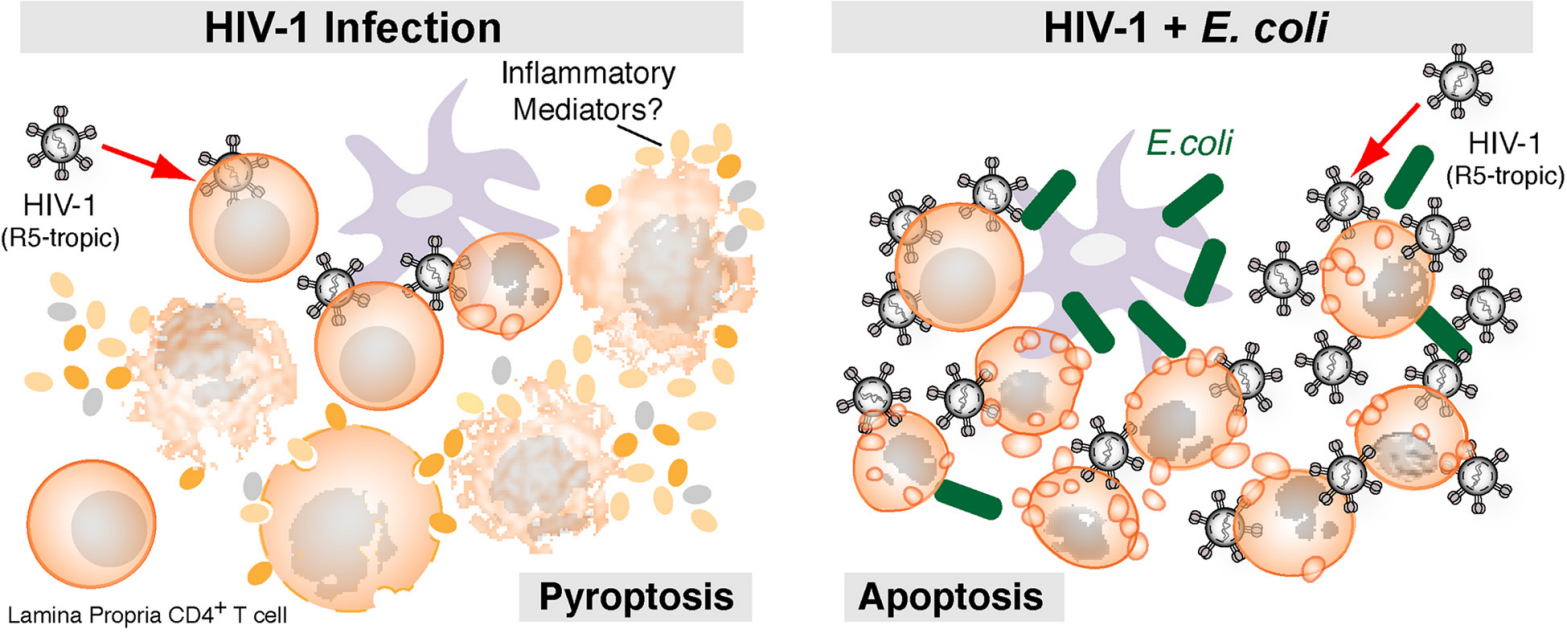
**Biphasic model of LP CD4+ T cell death during acute HIV-1 infection.** We theorize that in ‘early’ acute infection, prior to extensive microbial translation, pyroptotic death in bystander p24^neg^ cells could account for the majority of LP CD4+ T cell depletion. Inflammatory mediators released during pyroptosis may contribute to epithelial barrier break down. During ‘late’ acute HIV-1 infection, T cells and APCs exposed to commensal bacteria results in enhanced HIV-1 replication, and a shift in the LP CD4+ T cell death pathway to apoptosis. The loss of Th17 cells in particular could further exacerbate epithelial barrier dysfunction and contribute to a cycle of microbial entry, T cell activation, infection and death. Increased apoptotsis from productively infected cells could also contribute to the release of apoptotic microparticles into the periphery, which others have shown could reduce the potency of early adaptive immune responses against HIV-1.

Our findings in the *ex vivo* LPAC model have potential implications for understanding how HIV-1 induces LP CD4+ T cell depletion *in vivo*. Based on our *ex vivo* modeling results, we theorize that mucosal pathogenesis may proceed in two phases during early HIV-1 infection: (1) there could be an ‘early phase’ of depletion, before intestinal barrier breakdown occurs, when depletion and inflammation are simultaneously driven by Caspase-1 mediated PCD in uninfected LP CD4+ T cells; and (2) a ‘late phase’ that is driven by the onset of microbial translocation, with exposure to microbial products resulting in increased HIV-1 replication and a larger role for apoptotic death of infected LP CD4+ T cells in ongoing depletion (Figure 7). The potential *in vivo* consequences of apoptotic death of mucosal T cells could include a weakened adaptive immunity as highlighted by other investigators [47,48]. Corroborating this ‘biphasic model’ of early HIV-1 mucosal pathogenesis will require directly evaluating tissues from HIV-1 infected patients at varying disease stages. Further experiments in the LPAC model, informed by *in vivo* studies, should help refine mechanistic models of LP CD4+ T cell depletion and inflammation to ultimately provide a better understanding of HIV-1 mucosal pathogenesis.

## Conclusions

The data establish the Lamina Propria Aggregate Culture (LPAC) model as a robust and versatile platform for studying the impact of R5-tropic HIV-1 infection and commensal microbial species on mucosal CD4+ T cell death. Using the LPAC model, we provide evidence for Caspase-1 mediated T cell pyroptosis as a primary mechanism for LP CD4+ T cell death in bystander cells. However, upon exposure to commensal *E. coli*, LP CD4+ T cell death was augmented and shifted to Caspase-3 mediated apoptosis due to a significant increase in productively-infected cells. The results suggest a biphasic model of LP CD4+ T cell death during acute HIV infection, in which distinct CD4+ T cell death pathways, demarcated by intestinal barrier dysfunction and microbial translocation, may converge to drive mucosal inflammation, viral dissemination and systemic immune activation.

## Methods

### Intestinal tissue samples

Macroscopically normal human jejunum tissue samples, that would otherwise be discarded, were obtained in a de-identified manner from 17 patients undergoing elective abdominal surgery. Those with a history of inflammatory bowel disease, recent chemotherapy, radiation, or other immunosuppressive drugs were excluded from the study. The use of discarded tissue was granted exempt status by the Colorado Multiple Institutional Review Board and patients signed a pre-operative consent form allowing its unrestricted use.

### Preparation of LPMCs from intestinal samples

The primary LP cells were obtained using a two-step fractionation process as previously described [20,21]. EDTA was used to separate the epithelial cells and intraepithelial lymphocytes, followed by Collagenase D (Roche Catalog #: 11088882001) treatment to release the LPMCs. The cells were cryopreserved in RPMI + 10% DMSO + 10% FBS.

### Preparation of HIV-1 stocks

MOLT4-CCR5 cells (AIDS Research and Reference Reagent Program; ARRP Catalog# 510) were grown at 0.5 to 1 million (M)/ml in RPMI containing 10% FBS, 1% penicillin/streptomycin/glutamine and 1 mg/ml G418 (propagation media) at 37°C to 50 M total cells. Majority (>90%) of MOLT4-CCR5 cells expressed CD4 and CCR5 by flow cytometry. 25 M MOLT4-CCR5 cells were infected with the R5-tropic HIV-1 Ba-L strain (ARRP Catalog# 4984) with 2 μg/ml polybrene for 2 h. The cells were washed with propagation media and grown at 0.5 to 1 M cells/ml. Supernatants containing virus were collected at 9 dpi and concentrated by ultracentrifugation at 141,000×*g* for 2 h. The concentration of p24 in the supernatant was determined by HIV Gag p24 ELISA (Perkin Elmer, Walthm, MA). Virus stocks were frozen in single use aliquots at −80°C.

### Preparation of heat-killed *Eschericia coli*

*E. coli* stocks (ATCC #25922, Manassass, VA) were grown as described previously [21] and kept at −80°C in single use aliquots.

### LPMC infection assay

LPMCs were thawed using a standard protocol. Briefly, 1 ml aliquots of cells were quick thawed at 37°C. The following amounts of thaw media (90 ml RPMI + 10% FBS + 1% penicillin/streptomycin/glutamine + 100 μl DNAse) were added at 1 min intervals: 100 μl, 200 μl, 400 μl, 800 μl, 1.6 ml, 3.2 ml (twice). The cells were centrifuged at 1500 rpm for 5 mins and then washed once in 10 ml of thaw media. The thawed LPMCs were immediately resuspended in complete RPMI (RPMI + 10% human AB serum, 1% penicillin/streptomycin/glutamine, 500 μg/ml Zosyn) without any additional growth factors. HIV-1 (80 ng p24) per 1 M LPMCs was used to infect primary LPMCs at 10 M/ml in complete RPMI. LPMCs were mock infected in parallel. Infection by spinoculation was performed at 1200×*g* for 2 h at room temperature [49]. After spinoculation, the supernatant containing free virus was discarded; the cell pellet was resuspended in complete RPMI at 1 M LPMCs/ml and then seeded in a 48-well plate. Heat-killed *E. coli* was added to LPMCs at a ratio of 5 *E.coli*: 1 LPMC where indicated. Fungizone (1.25 μg/ml) was added at 1 dpi. The LPMCs were harvested at indicated time points.

### LPMC Infection in the presence of drugs

For infections in the presence of Efavirenz (ARRP Catalog #4624), LPMCs were pre-incubated with Efavirenz (1 nM or 10 nM) for 2 h at 37°C and infected as described above and cultured in media containing the indicated Efavirenz dose. A second dose of Efavirenz was added at 3 dpi. At 6 dpi, the LPMCs were collected and analyzed as described.

Inhibitors of Caspase-3 (DEVD-fmk; DEVD (Calbiochem)) and Caspase-1 (YVAD-cmk; YVAD (Calbiochem)) were used to inhibit PCD *ex vivo* in donor samples that showed depletion at 4 dpi. The YVAD motif is predominately cleaved by Caspase-1 but does have some cross reactivity (340-fold less affinity) with other group I caspases, Caspase-4 and Caspase-5 [50]. The inhibitors were resuspended in DMSO at 50 mM and stored at −20°C per the manufacturer’s instructions. The dose used in primary LPMCs was based on optimization studies to balance maximum inhibition of the pathway with minimum toxicity. The 4 dpi time point was chosen so that consistent depletion was observed but DMSO exposure was minimal. Following HIV-1 spinoculation (0 dpi), the inhibitors were added to the appropriate wells at a 25 μM concentration. An additional dose of each inhibitor was added to the cultures at 2 dpi without changing the media. At 4 dpi, LPMCs were collected for analysis.

### Mitogen Stimulation for Th Subset Identification

6 dpi LPMCs were collected, counted, resuspended at 1 M/ml in complete RPMI, and stimulated with 100 ng/ml PMA (Sigma-Aldrich), 100 μg/ml Ionomycin (Sigma- Aldrich) and 1 μg/ml Brefeldin A (Golgi Plug, BD Biosciences) for 5 h at 37°C in 5% CO_2_. LPMCs capable of making IFN-γ and IL-17 were identified by flow cytometry [21].

### Antibodies for flow cytometry

Aqua Viability Dye (AqVi; Invitrogen) exclusion was used to identify viable cells. The antibodies used for these studies were: CD3-ECD (Beckman Coulter), CD8-APC (BD Pharmingen), CD19-APCH7 (BD Biosciences), HIV-1 p24-PE (Beckman Coulter), IL-17-V450 (BD biosciences), IFN-γ-AF700 (BD biosciences) and AnnexinV-Pacific Blue (Life Technologies). Isotype controls were used to establish the gates for IL-17 (Mouse IgG1- V450, BD Biosciences) and IFNγ (Mouse IgG1-AF700, BD biosciences).

### Extracellular and Intracellular Staining

LPMCs were stained with cocktails of antibodies against markers expressed on the cell surface for 20 minutes in PBS + 1% BSA + 2 mM EDTA (FACS buffer) at 4°C. The LPMCs were then incubated with AqVi per the manufacturer’s instructions. The LPMCs were then fixed and permeabilized using the Invitrogen Medium A/Medium B system [21].

The best practices protocol from eBioscience was followed to detect AnnexinV + LPMCs in conjunction with intracellular markers incorporating minor modifications following the AqVi step in the standard protocol. After the LPMCs were incubated with AqVi, they were washed once with 2 ml of 1× AnnexinV binding buffer (BD Pharmingen). The LPMCs were resuspended in 1× AnnexinV binding buffer at a concentration of 1-3 M cells/ml and incubated, at room temperature for 15 min, with 5 μL of AnnexinV. Unbound AnnexinV was washed away using 2 ml 1× AnnexinV binding buffer. The cells were then fixed and stained for intracellular p24 using the Medium A/Medium B system. In optimization experiments, the early apoptotic phenotype was not detectable beyond 4 dpi. Therefore, we selected samples that consistently depleted at 4 dpi to determine the phenotype of the dying CD4+ T cells.

### Fluorogenic Detection of Caspase-1 Activity

2 dpi and 6 dpi LPMCs were collected and counted using trypan blue. The LPMCs were resuspended in 75 uL of 10 mM CaspaLux-1 substrate (Oncoimmunin) and incubated for 30 mins at 37°C in 5% CO_2_. The LPMCs were washed once with 1 mL of the provided FACS buffer and surface stained as described above. The LPMCs were fixed with 4% PFA for 15 mins at room temperature, washed once with 2 mL of FACS buffer, and acquired in 1% PFA on the LSRII within 60 mins.

### Flow cytometric acquisition and analysis

Acquisition occurred within 24 hours for all samples and within 2 h for samples stained with AnnexinV. All samples were collected on an LSR II flow cytometer (BD Biosciences) and analyzed using BD FACS DIVA version 6.1.3.

To quantify depletion, the number of viable T cells in an HIV-1 infected well was reported as a percent of the T cells in the mock infected control well at 4 or 6 dpi. The LPMCs were harvested and counted using trypan blue to determine the number of live cells in the well. However, because the LPAC model is a mixed cell culture model, the number of live cells counted by trypan blue does not equate to the number of viable CD4+ T cells per well. To determine the number of viable CD4+ T cells per well, flow cytometry was used to determine the percentage of the viable cells (analogous to the trypan blue count) that were CD3+CD8- in a tight lymphocyte gate that excluded both cellular debris and epithelial cells. The trypan blue count was then multiplied by the percentage of viable CD3+CD8- T cells measured by flow cytometry. For example, if 100,000 cells were counted by trypan blue and 40% were viable CD3+CD8- T cells by flow cytometry, we would report out 40,000 CD4+ T cells for that well. The same method was used to determine the number of Th1, Th17, Th1/17, and apoptotic or necrotic CD4+ T cells. CD4+ T cell depletion was determined in the presence and absence of *E.coli*.

### Statistical analysis

Statistical analysis was conducted using GraphPad Prism version 5 for Windows (GraphPad, San Diego, CA). Non-parametric statistics were used in these analyses.

## Competing interests

The authors declare they have no competing interests.

## Authors’ contributions

AKS participated in the conceptual design and execution of the experiments, analyzed the data, and helped draft the manuscript; EJL and JAM provided technical support and critically reviewed the manuscript; SMD and JDB critically read the manuscript and participated in conceptual discussions; MDM provided critical access to the surgical samples; MLS and CCW participated in the conceptual design of the studies and helped draft the manuscript. All authors read and approved the final manuscript.

## Supplementary Material

Additional file 1: Figure S1 LPMCs used to quantify LP CD4+ T cell survival following HIV-1 infection were phenotyped by flow cytometry prior to infection using the following antibodies: CD45- PerCP-cy5.5; HLA-DR- APC-Cy7; CD11c- PE-Cy5; BDCA-1- APC; CD3-ECD; CD4- AF700; CD8- PE; gdTCR- FITC; and AquaDye Viabilty dye (AqVi) (Invitrogen). Immune cell subsets were identified from within the CD45+ AqVi- population. The total dendritic cell population (HLA-DR+CD11c+) was divided into BDCA-1+ and BDCA-1- populations. The donors were divided into ‘depleters’ and ‘non-depleters’ as in Figure 1D. There were no significant differences observed in the LPMC composition at baseline between donors that depleted 4 dpi and donors that did not. Significance was determined using the Wilcoxon matched-pairs signed rank test within each phenotype. In all cases *p* > 0.05. Click here for file

Additional file 2: Figure S2 AnnexinV binding to exposed phosphotidylserine on the cell surface and uptake of AqVi was measured on p24+ versus p24^neg^ LP CD3+CD8- T cells at 4 dpi. Each symbol is a unique donor. The horizontal line indicates the median value and the fold-difference between median values is indicated. Statistical significance was determined using Wilcoxon matched-paired signed rank test. *, *p* = 0.02; **, *p* = 0.002. **(A)** The percentage of p24+ and p24^neg^ cells that have either an apoptotic (*left*) or necrotic (*right*) phenotype during HIV-1 infection *ex vivo*. **(B)** The percentage of p24+ and p24^neg^ cells that have either an apoptotic (*left*) or necrotic (*right*) phenotype in the presence of *E.coli*. Click here for file

Additional file 3: Figure S3 LPMCs were infected with HIV-1_Ba-L_ in the presence or absence of *E.coli* for 4 days. Irreversible inhibitors of Caspase-1 (YVAD) and Caspase-3 (DEVD) at 25 μM were used to block caspase function. DMSO was used as a vehicle control. Mock infections ± inhibitors and DMSO were established in parallel and used to set the p24 gate. **(A)** The percent of p24+ cells at 4 dpi in the absence of *E. coli.* Each symbol is a unique donor. The median infection frequency is shown as the horizontal line. No significant differences were identified using a non-parametric repeated measures ANOVA, *p* = 0.43. **(B)** The percentage of p24+ cells in the presence of *E.coli* as described in panel **A**. Overall ANOVA, *p* = 0.44. Note that *E.coli* exposure enhanced HIV-1 infection to a similar extent in the presence or absence of caspase inhibitors (compare panel **B** to **A**). **(C and D)** Inhibitors do not impact the total cell number in mock + *E.coli* conditions. The absolute number of LP CD4+ T cells in the mock + *E.coli* condition in the presence of **(C)** DEVD or **(D)** YVAD. Each donor is represented by a unique symbol. The horizontal lines indicate the median cell number. Using Wilcoxon matched-paired signed rank test, no significant differences were observed in panels **C** and **D**, *p* > 0.05. Click here for file

## References

[B1] Brenchley JM Schacker TW Ruff LE Price DA Taylor JH Beilman GJ Nguyen PL Khoruts A Larson M Haase AT Douek DC CD4+ T cell depletion during all stages of HIV disease occurs predominantly in the gastrointestinal tract J Exp Med 2004 200 749 759 10.1084/jem.20040874 15365096 PMC2211962

[B2] Sankaran S Guadalupe M Reay E George MD Flamm J Prindiville T Dandekar S Gut mucosal T cell responses and gene expression correlate with protection against disease in long-term HIV-1-infected nonprogressors Proc Natl Acad Sci U S A 2005 102 9860 9865 10.1073/pnas.0503463102 15980151 PMC1159164

[B3] Chase A Zhou Y Siliciano RF HIV-1-induced depletion of CD4+ T cells in the gut: mechanism and therapeutic implications Trends Pharmacol Sci 2006 27 4 7 10.1016/j.tips.2005.11.005 16337692

[B4] Thomas C Roadblocks in HIV research: five questions Nat Med 2009 15 855 859 10.1038/nm0809-855 19661992

[B5] Doitsh G Cavrois M Lassen KG Zepeda O Yang Z Santiago ML Hebbeler AM Greene WC Abortive HIV infection mediates CD4 T cell depletion and inflammation in human lymphoid tissue Cell 2010 143 789 801 10.1016/j.cell.2010.11.001 21111238 PMC3026834

[B6] Cooper A Garcia M Petrovas C Yamamoto T Koup RA Nabel GJ HIV-1 causes CD4 cell death through DNA-dependent protein kinase during viral integration Nature 2013 498 376 379 10.1038/nature12274 23739328

[B7] Mattapallil JJ Douek DC Hill B Nishimura Y Martin M Roederer M Massive infection and loss of memory CD4+ T cells in multiple tissues during acute SIV infection Nature 2005 434 1093 1097 10.1038/nature03501 15793563

[B8] Li Q Duan L Estes JD Ma ZM Rourke T Wang Y Reilly C Carlis J Miller CJ Haase AT Peak SIV replication in resting memory CD4+ T cells depletes gut lamina propria CD4+ T cells Nature 2005 434 1148 1152 15793562 10.1038/nature03513

[B9] Kunkel EJ Boisvert J Murphy K Vierra MA Genovese MC Wardlaw AJ Greenberg HB Hodge MR Wu L Butcher EC Campbell JJ Expression of the chemokine receptors CCR4, CCR5, and CXCR3 by human tissue-infiltrating lymphocytes Am J Pathol 2002 160 347 355 10.1016/S0002-9440(10)64378-7 11786428 PMC1867126

[B10] Wilkin TJ Su Z Kuritzkes DR Hughes M Flexner C Gross R Coakley E Greaves W Godfrey C Skolnik PR HIV type 1 chemokine coreceptor use among antiretroviral-experienced patients screened for a clinical trial of a CCR5 inhibitor: AIDS Clinical Trial Group A5211 Clin Infect Dis 2007 44 591 595 10.1086/511035 17243065

[B11] Keele BF Giorgi EE Salazar-Gonzalez JF Decker JM Pham KT Salazar MG Sun C Grayson T Wang S Li H Identification and characterization of transmitted and early founder virus envelopes in primary HIV-1 infection Proc Natl Acad Sci U S A 2008 105 7552 7557 10.1073/pnas.0802203105 18490657 PMC2387184

[B12] Smith PM Garrett WS The gut microbiota and mucosal T cells Front Microbiol 2011 2 111 21833339 10.3389/fmicb.2011.00111 PMC3153059

[B13] Brenchley JM Paiardini M Knox KS Asher AI Cervasi B Asher TE Scheinberg P Price DA Hage CA Kholi LM Differential Th17 CD4 T-cell depletion in pathogenic and nonpathogenic lentiviral infections Blood 2008 112 2826 2835 10.1182/blood-2008-05-159301 18664624 PMC2556618

[B14] Ciccone EJ Read SW Mannon PJ Yao MD Hodge JN Dewar R Chairez CL Proschan MA Kovacs JA Sereti I Cycling of gut mucosal CD4+ T cells decreases after prolonged anti-retroviral therapy and is associated with plasma LPS levels Mucosal Immunol 2010 3 172 181 10.1038/mi.2009.129 19956090 PMC2830855

[B15] Favre D Mold J Hunt PW Kanwar B Loke P Seu L Barbour JD Lowe MM Jayawardene A Aweeka F Tryptophan catabolism by indoleamine 2,3-dioxygenase 1 alters the balance of TH17 to regulatory T cells in HIV disease Sci Transl Med 2010 2 32 a36 10.1126/scitranslmed.3000632 PMC3034445 20484731

[B16] Brenchley JM Price DA Schacker TW Asher TE Silvestri G Rao S Kazzaz Z Bornstein E Lambotte O Altmann D Microbial translocation is a cause of systemic immune activation in chronic HIV infection Nat Med 2006 12 1365 1371 17115046 10.1038/nm1511

[B17] Jiang W Lederman MM Hunt P Sieg SF Haley K Rodriguez B Landay A Martin J Sinclair E Asher AI Plasma levels of bacterial DNA correlate with immune activation and the magnitude of immune restoration in persons with antiretroviral-treated HIV infection J Infect Dis 2009 199 1177 1185 10.1086/597476 19265479 PMC2728622

[B18] Pandrea I Gaufin T Brenchley JM Gautam R Monjure C Gautam A Coleman C Lackner AA Ribeiro RM Douek DC Apetrei C Cutting edge: experimentally induced immune activation in natural hosts of simian immunodeficiency virus induces significant increases in viral replication and CD4+ T cell depletion J Immunol 2008 181 6687 6691 18981083 10.4049/jimmunol.181.10.6687 PMC2695139

[B19] Estes JD Harris LD Klatt NR Tabb B Pittaluga S Paiardini M Barclay GR Smedley J Pung R Oliveira KM Damaged intestinal epithelial integrity linked to microbial translocation in pathogenic simian immunodeficiency virus infections PLoS Pathog 2010 6 e1001052 10.1371/journal.ppat.1001052 20808901 PMC2924359

[B20] Howe R Dillon S Rogers L McCarter M Kelly C Gonzalez R Madinger N Wilson CC Evidence for dendritic cell-dependent CD4(+) T helper-1 type responses to commensal bacteria in normal human intestinal lamina propria Clin Immunol 2009 131 317 332 10.1016/j.clim.2008.12.003 19174326 PMC2720400

[B21] Dillon SM Manuzak JA Leone AK Lee EJ Rogers LM McCarter MD Wilson CC HIV-1 infection of human intestinal lamina propria CD4+ T cells in vitro is enhanced by exposure to commensal Escherichia coli J Immunol 2012 189 885 896 10.4049/jimmunol.1200681 22689879 PMC3395168

[B22] Kroemer G Galluzzi L Vandenabeele P Abrams J Alnemri ES Baehrecke EH Blagosklonny MV El-Deiry WS Golstein P Green DR Classification of cell death: recommendations of the Nomenclature Committee on Cell Death 2009 Cell Death Differ 2009 16 3 11 10.1038/cdd.2008.150 18846107 PMC2744427

[B23] Galluzzi L Vitale I Abrams JM Alnemri ES Baehrecke EH Blagosklonny MV Dawson TM Dawson VL El-Deiry WS Fulda S Molecular definitions of cell death subroutines: recommendations of the Nomenclature Committee on Cell Death 2012 Cell Death Differ 2012 19 107 120 10.1038/cdd.2011.96 21760595 PMC3252826

[B24] Fink SL Cookson BT Apoptosis, pyroptosis, and necrosis: mechanistic description of dead and dying eukaryotic cells Infect Immun 2005 73 1907 1916 10.1128/IAI.73.4.1907-1916.2005 15784530 PMC1087413

[B25] Silva MT do Vale A dos Santos NM Secondary necrosis in multicellular animals: an outcome of apoptosis with pathogenic implications Apoptosis 2008 13 463 482 10.1007/s10495-008-0187-8 18322800 PMC7102248

[B26] Opferman JT Korsmeyer SJ Apoptosis in the development and maintenance of the immune system Nat Immunol 2003 4 410 415 10.1038/ni0503-410 12719730

[B27] Alimonti JB Ball TB Fowke KR Mechanisms of CD4+ T lymphocyte cell death in human immunodeficiency virus infection and AIDS J Gen Virol 2003 84 1649 1661 10.1099/vir.0.19110-0 12810858

[B28] Arnoult D Viollet L Petit F Lelievre JD Estaquier J HIV-1 triggers mitochondrion death Mitochondrion 2004 4 255 269 10.1016/j.mito.2004.06.010 16120390

[B29] Gougeon ML Piacentini M New insights on the role of apoptosis and autophagy in HIV pathogenesis Apoptosis 2009 14 501 508 10.1007/s10495-009-0314-1 19199038

[B30] Miao EA Rajan JV Aderem A Caspase-1-induced pyroptotic cell death Immunol Rev 2011 243 206 214 10.1111/j.1600-065X.2011.01044.x 21884178 PMC3609431

[B31] Bergsbaken T Fink SL Cookson BT Pyroptosis: host cell death and inflammation Nat Rev Microbiol 2009 7 99 109 10.1038/nrmicro2070 19148178 PMC2910423

[B32] Al-Sadi R Ye D Said HM Ma TY Cellular and molecular mechanism of interleukin-1beta modulation of Caco-2 intestinal epithelial tight junction barrier J Cell Mol Med 2011 15 970 982 10.1111/j.1582-4934.2010.01065.x 20406328 PMC3922681

[B33] Roselli M Finamore A Britti MS Mengheri E Probiotic bacteria Bifidobacterium animalis MB5 and Lactobacillus rhamnosus GG protect intestinal Caco-2 cells from the inflammation-associated response induced by enterotoxigenic Escherichia coli K88 Br J Nutr 2006 95 1177 1184 10.1079/BJN20051681 16768842

[B34] Scheuring UJ Sabzevari H Corbeil J Theofilopoulos AN Differential expression profiles of apoptosis-affecting genes in HIV-infected cell lines and patient T cells AIDS 1999 13 167 175 10.1097/00002030-199902040-00004 10202822

[B35] Sloand EM Kumar PN Kim S Chaudhuri A Weichold FF Young NS Human immunodeficiency virus type 1 protease inhibitor modulates activation of peripheral blood CD4(+) T cells and decreases their susceptibility to apoptosis in vitro and in vivo Blood 1999 94 1021 1027 10419894

[B36] Rhee SS Marsh JW Human immunodeficiency virus type 1 Nef-induced down-modulation of CD4 is due to rapid internalization and degradation of surface CD4 J Virol 1994 68 5156 5163 8035515 10.1128/jvi.68.8.5156-5163.1994 PMC236459

[B37] Vanden Berghe T Vanlangenakker N Parthoens E Deckers W Devos M Festjens N Guerin CJ Brunk UT Declercq W Vandenabeele P Necroptosis, necrosis and secondary necrosis converge on similar cellular disintegration features Cell Death Differ 2010 17 922 930 10.1038/cdd.2009.184 20010783

[B38] Favre D Lederer S Kanwar B Ma ZM Proll S Kasakow Z Mold J Swainson L Barbour JD Baskin CR Critical loss of the balance between Th17 and T regulatory cell populations in pathogenic SIV infection PLoS Pathog 2009 5 e1000295 10.1371/journal.ppat.1000295 19214220 PMC2635016

[B39] Gosselin A Monteiro P Chomont N Diaz-Griffero F Said EA Fonseca S Wacleche V El-Far M Boulassel MR Routy JP Peripheral blood CCR4 + CCR6+ and CXCR3 + CCR6 + CD4+ T cells are highly permissive to HIV-1 infection J Immunol 2010 184 1604 1616 10.4049/jimmunol.0903058 20042588 PMC4321756

[B40] Monteiro P Gosselin A Wacleche VS El-Far M Said EA Kared H Grandvaux N Boulassel MR Routy JP Ancuta P Memory CCR6 + CD4+ T cells are preferential targets for productive HIV type 1 infection regardless of their expression of integrin beta7 J Immunol 2011 186 4618 4630 10.4049/jimmunol.1004151 21398606

[B41] Doitsh G Galloway NL Geng X Yang Z Monroe KM Zepeda O Hunt PW Hatano H Sowinski S Munoz-Arias I Greene WC Cell death by pyroptosis drives CD4 T-cell depletion in HIV-1 infection Nature 2013 505 509 514 10.1038/nature12940 24356306 PMC4047036

[B42] Monroe KM Yang Z Johnson JR Geng X Doitsh G Krogan NJ Greene WC IFI16 DNA sensor is required for death of lymphoid CD4 T cells abortively infected with HIV Science 2013 343 428 432 24356113 10.1126/science.1243640 PMC3976200

[B43] Aziz S Fackler OT Meyerhans A Muller-Lantzsch N Zeitz M Schneider T Replication of M-tropic HIV-1 in activated human intestinal lamina propria lymphocytes is the main reason for increased virus load in the intestinal mucosa J Acquir Immune Defic Syndr 2005 38 23 30 10.1097/00126334-200501010-00005 15608520

[B44] Nazli A Chan O Dobson-Belaire WN Ouellet M Tremblay MJ Gray-Owen SD Arsenault AL Kaushic C Exposure to HIV-1 directly impairs mucosal epithelial barrier integrity allowing microbial translocation PLoS Pathog 2010 6 e1000852 10.1371/journal.ppat.1000852 20386714 PMC2851733

[B45] Fallarino F Grohmann U Vacca C Bianchi R Orabona C Spreca A Fioretti MC Puccetti P T cell apoptosis by tryptophan catabolism Cell Death Differ 2002 9 1069 1077 10.1038/sj.cdd.4401073 12232795

[B46] Lee GK Park HJ Macleod M Chandler P Munn DH Mellor AL Tryptophan deprivation sensitizes activated T cells to apoptosis prior to cell division Immunology 2002 107 452 460 10.1046/j.1365-2567.2002.01526.x 12460190 PMC1782830

[B47] Frleta D Ochoa CE Kramer HB Khan SA Stacey AR Borrow P Kessler BM Haynes BF Bhardwaj N HIV-1 infection-induced apoptotic microparticles inhibit human DCs via CD44 J Clin Invest 2012 122 4685 4697 10.1172/JCI64439 23160198 PMC3533550

[B48] Gasper-Smith N Crossman DM Whitesides JF Mensali N Ottinger JS Plonk SG Moody MA Ferrari G Weinhold KJ Miller SE Induction of plasma (TRAIL), TNFR-2, Fas ligand, and plasma microparticles after human immunodeficiency virus type 1 (HIV-1) transmission: implications for HIV-1 vaccine design J Virol 2008 82 7700 7710 10.1128/JVI.00605-08 18508902 PMC2493338

[B49] O’Doherty U Swiggard WJ Malim MH Human immunodeficiency virus type 1 spinoculation enhances infection through virus binding J Virol 2000 74 10074 10080 10.1128/JVI.74.21.10074-10080.2000 11024136 PMC102046

[B50] Garcia-Calvo M Peterson EP Leiting B Ruel R Nicholson DW Thornberry NA Inhibition of human caspases by peptide-based and macromolecular inhibitors J Biol Chem 1998 273 32608 32613 10.1074/jbc.273.49.32608 9829999

