# Supplementary figures and images for: Microbial exposure alters HIV-1-induced mucosal CD4+ T cell death pathways Ex vivo
Source: Retrovirology. 2014 Feb 4;11:14. doi: 10.1186/1742-4690-11-14 (PMC3922902; doi:10.1186/1742-4690-11-14)

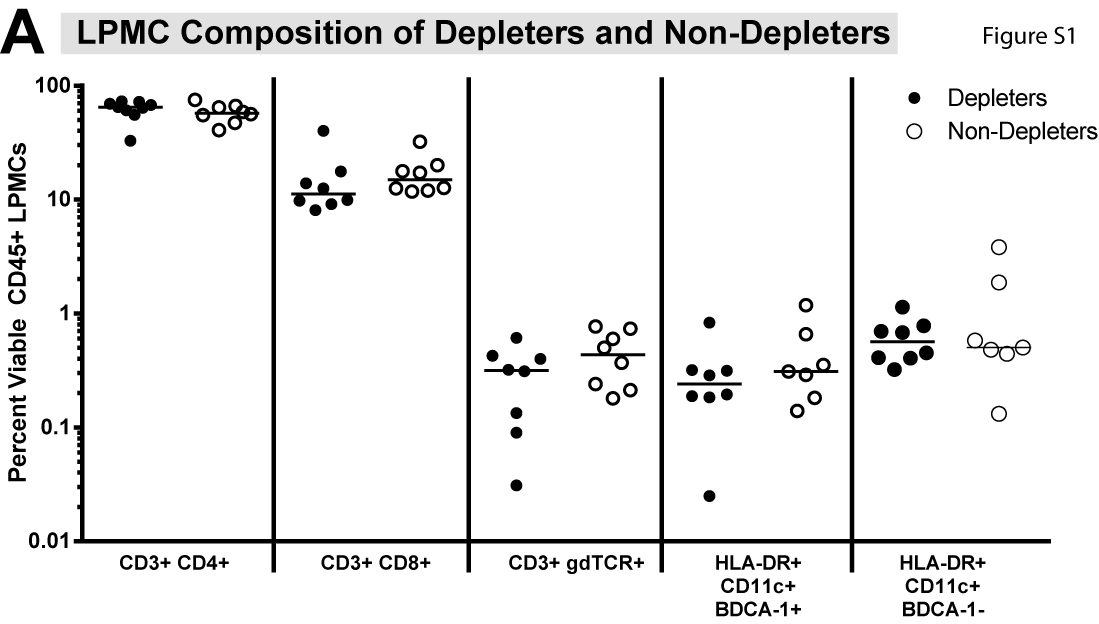

Supplement: Additional file 1: Figure S1 — LPMCs used to quantify LP CD4+ T cell survival following HIV-1 infection were phenotyped by flow cytometry prior to infection using the following antibodies: CD45- PerCP-cy5.5; HLA-DR- APC-Cy7; CD11c- PE-Cy5; BDCA-1- APC; CD3-ECD; CD4- AF700; CD8- PE; gdTCR- FITC; and AquaDye Viabilty dye (AqVi) (Invitrogen). Immune cell subsets were identified from within the CD45+ AqVi- population. The total dendritic cell population (HLA-DR+CD11c+) was divided into BDCA-1+ and BDCA-1- populations. The donors were divided into ‘depleters’ and ‘non-depleters’ as in Figure 1D. There were no significant differences observed in the LPMC composition at baseline between donors that depleted 4 dpi and donors that did not. Significance was determined using the Wilcoxon matched-pairs signed rank test within each phenotype. In all cases p > 0.05. [file 1742-4690-11-14-S1.tiff]

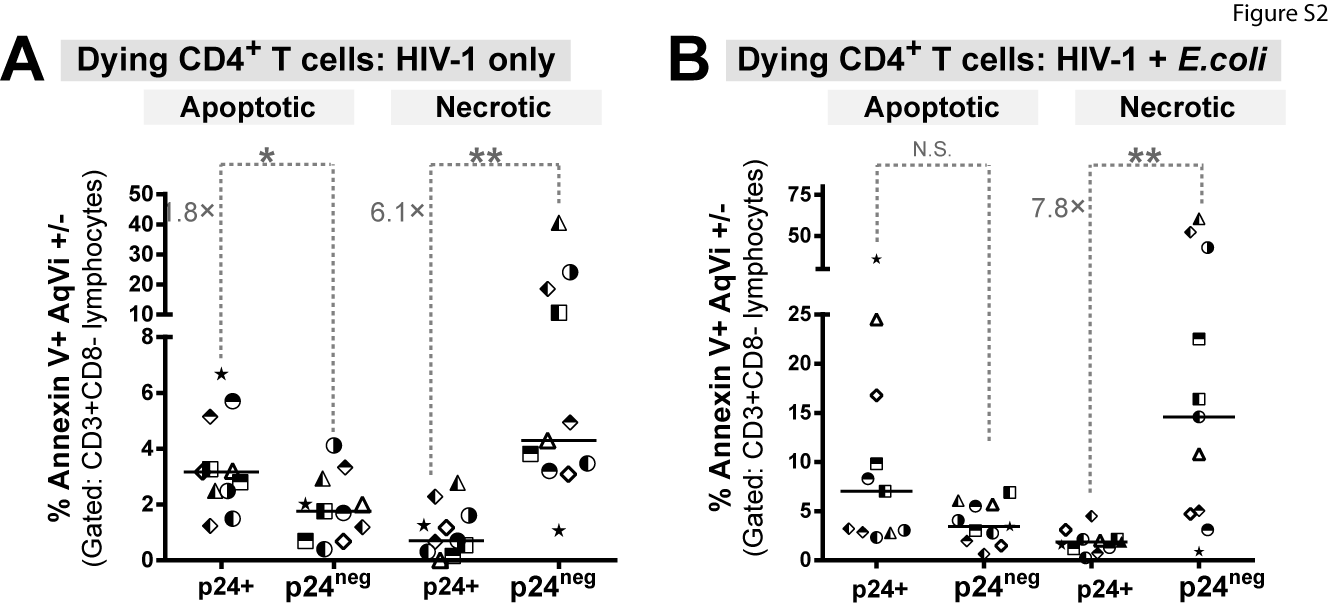

Supplement: Additional file 2: Figure S2 — AnnexinV binding to exposed phosphotidylserine on the cell surface and uptake of AqVi was measured on p24+ versus p24neg LP CD3+CD8- T cells at 4 dpi. Each symbol is a unique donor. The horizontal line indicates the median value and the fold-difference between median values is indicated. Statistical significance was determined using Wilcoxon matched-paired signed rank test. *, p = 0.02; **, p = 0.002. (A) The percentage of p24+ and p24neg cells that have either an apoptotic (left) or necrotic (right) phenotype during HIV-1 infection ex vivo. (B) The percentage of p24+ and p24neg cells that have either an apoptotic (left) or necrotic (right) phenotype in the presence of E.coli. [file 1742-4690-11-14-S2.tiff]

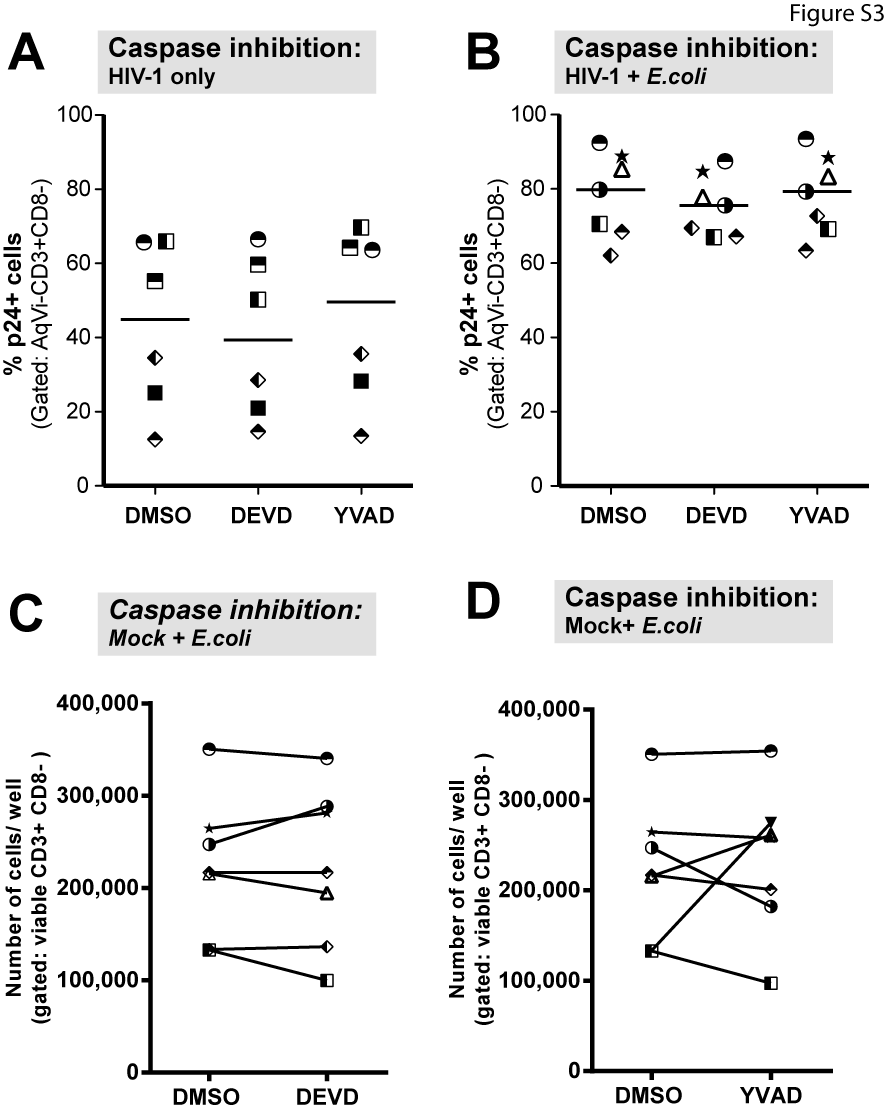

Supplement: Additional file 3: Figure S3 — LPMCs were infected with HIV-1Ba-L in the presence or absence of E.coli for 4 days. Irreversible inhibitors of Caspase-1 (YVAD) and Caspase-3 (DEVD) at 25 μM were used to block caspase function. DMSO was used as a vehicle control. Mock infections ± inhibitors and DMSO were established in parallel and used to set the p24 gate. (A) The percent of p24+ cells at 4 dpi in the absence of E. coli. Each symbol is a unique donor. The median infection frequency is shown as the horizontal line. No significant differences were identified using a non-parametric repeated measures ANOVA, p = 0.43. (B) The percentage of p24+ cells in the presence of E.coli as described in panel A. Overall ANOVA, p = 0.44. Note that E.coli exposure enhanced HIV-1 infection to a similar extent in the presence or absence of caspase inhibitors (compare panel B to A). (C and D) Inhibitors do not impact the total cell number in mock + E.coli conditions. The absolute number of LP CD4+ T cells in the mock + E.coli condition in the presence of (C) DEVD or (D) YVAD. Each donor is represented by a unique symbol. The horizontal lines indicate the median cell number. Using Wilcoxon matched-paired signed rank test, no significant differences were observed in panels C and D, p > 0.05. [file 1742-4690-11-14-S3.tiff]
